# BARD1 mystery: tumor suppressors are cancer susceptibility genes

**DOI:** 10.1186/s12885-022-09567-4

**Published:** 2022-06-01

**Authors:** Yousef M. Hawsawi, Anwar Shams, Abdulrahman Theyab, Wed A. Abdali, Nahed A. Hussien, Hanan E. Alatwi, Othman R. Alzahrani, Atif Abdulwahab A. Oyouni, Ahmad O. Babalghith, Mousa Alreshidi

**Affiliations:** 1 King Faisal Specialist Hospital and Research Center- Research Center, KFSH&RC, MBC-J04, P.O. Box 40047, Jeddah, 21499 Saudi Arabia; 2 College of Medicine, Al-Faisal University, P.O. Box 50927, Riyadh, 11533 Saudi Arabia; 3 Department of Pharmacology, College of Medicine, Taif University, P.O. Box 11099, Taif, 21944 Saudi Arabia; 4 Department of Laboratory Medicine, Security Forces Hospital, Mecca, Kingdom of Saudi Arabia; 5 Department of Zoology, Faculty of Science, Cairo University, Giza, 12613 Egypt; 6 Department of Biology, College of Science, Taif University, P.O Box 11099, Taif, 21944 Saudi Arabia; 7 Department of Biology, Faculty of Sciences, University of Tabuk, Tabuk, Kingdom of Saudi Arabia; 8 Genome and Biotechnology Unit, Faculty of Science, University of Tabuk, Tabuk, Saudi Arabia; 9 Medical genetics Department, College of Medicine, Umm Alqura University, Makkah, Saudi Arabia; 10 Departement of biology, College of Science, University of Hail, Hail, Saudi Arabia; 11 Molecular Diagnostic and Personalized Therapeutic Unit, University of Hail, Hail, Saudi Arabia

**Keywords:** BARD1, Breast Cancer, Tumor suppressor, Oncogene

## Abstract

The full-length BRCA1-associated RING domain 1 (BARD1) gene encodes a 777-aa protein. BARD1 displays a dual role in cancer development and progression as it acts as a tumor suppressor and an oncogene. Structurally, BARD1 has homologous domains to BRCA1 that aid their heterodimer interaction to inhibit the progression of different cancers such as breast and ovarian cancers following the BRCA1-dependant pathway. In addition, BARD1 was shown to be involved in other pathways that are involved in tumor suppression (BRCA1-independent pathway) such as the TP53-dependent apoptotic signaling pathway. However, there are abundant BARD1 isoforms exist that are different from the full-length BARD1 due to nonsense and frameshift mutations, or deletions were found to be associated with susceptibility to various cancers including neuroblastoma, lung, breast, and cervical cancers. This article reviews the spectrum of BARD1 full-length genes and its different isoforms and their anticipated associated risk. Additionally, the study also highlights the role of BARD1 as an oncogene in breast cancer patients and its potential uses as a prognostic/diagnostic biomarker and as a therapeutic target for cancer susceptibility testing and treatment.

## Introduction

**Fig. 1:**
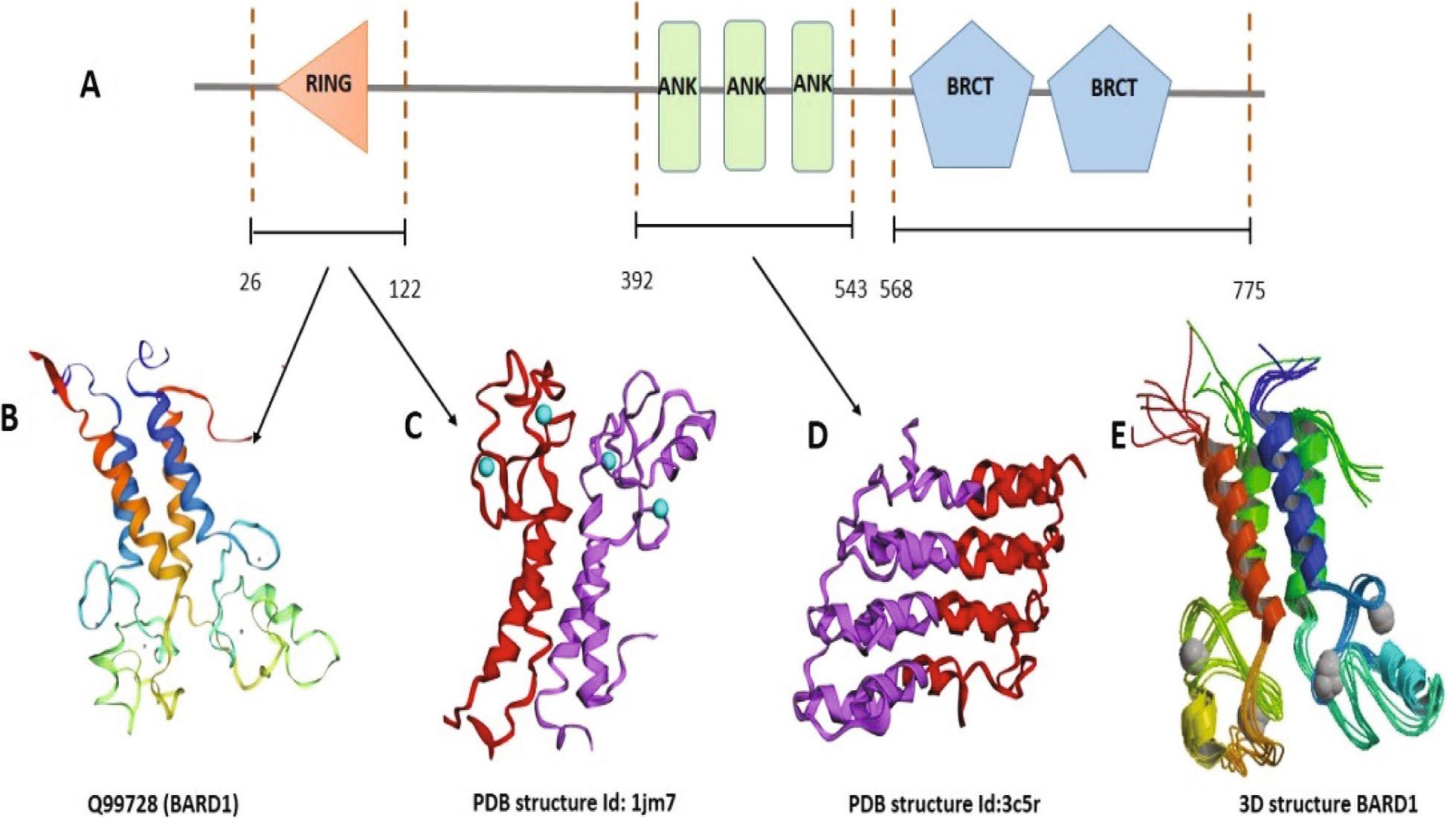
The 3D model of BARD1 gene structure from in-silico analysis by different online modeling tools: **A** Diagram showing the domanial arrangement of BARD1 gene. **B** homology model for the BARD1 gene created by SWISS-MODEL (Q99728/1JM7B). **C** The PDB structure Id: 1jm7. **D** The PDB structure Id: Id:3c5r. **E** 3D homology model for the BARD1 gene

In recent decades, researchers have studied the role of the BARD1 (BRCA1-associated RING domain 1) gene in cancer progression and its usage as a prognostic biomarker for cancer and a potential candidate for targeted cancer therapy [1]. The human BARD1 gene is located in chromosome 2 (2q34–35) and consists of 11 exons that encode a 777 aa protein with a molecular weight of 87 kDa. Structurally, BARD1 protein consists of a RING-finger domain at the N-terminal region, an intervening three repeated domains of Ankyrin (ANK), followed by two tandems of BRCA1 domains at the C-terminal region (BRCT) (Fig. 1A) [2]. These BRCT repeats play an essential role in regulating the interactions with other partners’ proteins in a phosphorylation-based approach. These interactors proteins are required to mediate cellular processes such as DNA-damage checkpoint, DNA-repair machinery, and cell cycle regulation [3, 4]. Notably, both the RING-finger domain and BRCT repeats are fundamental for the onco-suppression effect of the BRCA1-BARD1 complex [5, 6].

In addition to the full-length BARD1 (FL-BARD1), there are different BARD1 isoforms with skipped exons and varied molecular weights [2]. The isoforms are more abundantly associated with cancer cells [7–9]. Isoform α lacks exon 2, while the β isoform lacks exons 2 and 3, leading to translation of shorter proteins (758 aa (85 kDa) and 680 aa (75 kDa), respectively) due to a frameshift of the open reading frame (ORF). Isoform γ was disrupted by exon four deletion, and isoforms φ and δ produce 326aa (37 kDa) and 307 aa (35 kDa) proteins because they are missing exons 2–6 and 3–6, respectively. The absence of exons 4–9 produces isoform ε with a molecular weight of 30 kDa (264 aa), while the lack of exons 1, 10, and 11 creates isoform η. Any other splicing from exons 1 to 10 disrupts the ORF. Therefore, the start codon of translation could occur at other alternative ORFs, resulting in a short protein of 167 aa (19 kDa). Interestingly, most of these isoforms were reported to have agonistic cancer susceptibility potential due to their lack of the RING finger and/or ankyrin repeats, which are required for the tumor suppressor functions of the FL- BARD1 [10, 11].

Functionally, BARD1 protein acts as a tumor suppressor in BRCA1-dependent/independent pathways. Formation of the BRCA1/BARD1 heterodimer (due to their homologous domains) via the N-terminal RING-finger domains (Fig. 1B-E) affects the activity of ubiquitin ligase, which participates in DNA damage response pathways, regulation of the cell cycle, and modulation of chromatin structure and hormone signaling [12, 13]. In cancer cells, mutations that disrupt the BRCA1-BARD1 heterodimers ensue detrimental degradation of both proteins [14, 15].

### BARD1 as a tumor-suppressor gene

The advancement of technology revealed the critical role of many genes, such as BRCA1/2 and BARD1, in hereditary and familial breast and ovarian cancers [16–18]. Currently, science has determined the anti-breast cancer role of BARD1 in the BRCA1- dependent pathway. BARD1-BRCA1 complex plays an essential role in DNA damage machinery as it induces ubiquitination via E3 ubiquitin ligase activity and degradation of the damaged proteins [12]. Westermark et al. [19] report the participation of BARD1 and BRCA1 in a homology-directed repair (HDR) of chromosomal breaks that clarify their presence with RAD51 in response to DNA damage [20, 21]. Additionally, the BARD1 BRCT domain facilitates the early recruitment of the BRCA1/BARD1 heterodimer to DNA damage sites through a specific interaction with poly (ADP-ribose) polymerase (PARP) [22]. Studies have also shown that disrupting mutations in the phosphate-binding pocket of the BARD1 BRCT domain in mice (S563F and K607A) inhibits the recruitment of the BRCA1/BARD1 heterodimer to the stalled replication fork (SRF), which leads to nucleolytic degradation of the SRF and eventually induces chromosomal instability [23]. These mutations have no impact on recruitment to HDR [23], as opposed to the comparable mutation in BRCA1 BRCT (S1598F) [6]. Furthermore, the BRCA1/BARD1 heterodimer interaction was disrupted by BARD1 or BRACA1 mutations associated with the presence of breast cancer, such as mutations of the RING finger domain [24, 25], missense mutations [26–28], and alterations of ANK sequences that are involved in the regulation of transcription [29]. In addition, the heterodimer, with the help of cleavage stimulation factor subunit 1 (CSTF1), inhibits inappropriate mRNA polyadenylation at DNA repair sites [30, 31]. BRCA1/BARD1 also contributes to tumor suppression via the ubiquitination pathway [32] and subcellular localization of BRCA1 [33].

BARD1 also acts as a tumor suppressor in a BRCA1- independent pathway as it interacts with the repeated sequences of the BCL3 ankyrin domains and in turn modulates the activities of transcription factor NFKB in the TP53-dependent apoptotic signaling pathway [34, 35]. In addition, a decrease in BARD1 protein expression has been associated with cellular changes linked to a premalignant phenotype [36]. Moreover, BARD1 and BRCA1 null mice were nearly similar in their phenotype, and BARD1 has a role in genomic integrity maintenance. Loss of BARD1 leads to chromosomal instability and embryonic death in the early stages [37]. Additionally, BARD1 was found to promote the ubiquitination of RNA polymerase II and prevent transcription of damaged DNA and ubiquitination of ER-alpha and beta that play a role in cellular proliferation during breast cancer development [38]. All these functions support the tumor suppressor role of the FL-BARD1 in contrast, BARD1 isoforms such as BARD1 β and BARD1 δ were reported to oppose this function and further promote cancer progression [39].

Recently, an association of the BARD1 gene with ovarian cancer was suggested in the Exome Sequencing Project and Exome Aggregation Consortium for 1915 patients [40] with a mutation frequency of 0.2% for the BARD1 gene. Compelling evidence have shown an association between BARD1 mutations and breast and/or ovarian cancer susceptibility; consequently, BARD1 is now included in panels of clinical genes testing for cancer susceptibility [41].

### BARD1 as an oncogene

Approximately 19 different expressed isoforms of BARD1 have been previously identified [7, 42], and some of these are reported to have an oncogenic function, such as BARD1β, κ, and π [7, 8]. While the FL- BARD1, either individually or in complex with BRCA1, was reported to display a tumor suppressor function [39], BARD1 isoforms such as BARD1β and δ have an antagonistic effect on full-length BARD1, leading to cancer susceptibility and oncogenicity [7]. Most isoforms of BARD1 have BRCT domains but lack the RING finger domain needed for BRCA1 heterodimer formation. Abnormal BARD1 isoforms are found in non-small cell lung cancer (NSCLC), breast, colon, and ovarian cancers delivering a role in cancer tumorigenesis and progression. In addition, it was reported that BARD1 isoforms expression is significantly associated with a decrease in the survival rate of cancer patients [7, 11].

Abnormalities of BARD1 isoforms are due to their protein translation from an alternative open reading frame (ORF). For instance, BARD1 γ can be translated starting from exon 3, followed by the translation of exons 4 through 11 as a noncontinuous ORF. In addition, BARD1 isoforms have been shown to antagonize the BRCA1-BARD1 ubiquitin ligase activity essential to induce cancer cell decease [7, 11, 38]. Furthermore, the expression of BARD1β has been associated with impaired homologous recombination (HR) and negatively impacted ubiquitin ligase activity in PARPi-sensitive colon cancer cells [43].

#### Impacts of epigenetic regulation on BARD1 gene expression and biological consequences

BARD1, as a BRCA1 heterodimer interactor that plays an essential role as a tumor suppressor gene altered in breast and ovarian cancers, was also found to display a BRCA1-independent function during cancer progression. BARD1 isoforms, including exon 6 to exon 11 (truncated isoforms), were highly expressed in acute myeloid leukemia (AML) ex vivo blasts compared to FL- BARD1 expression level. Lepore et al. showed that treatment of AML cells, MCF-7 breast cancer cells, and Kelly neuroblastoma cells with HDACi (Vorinostat) regulate epigenetically BARD1 mRNA expression. Upon Vorinostat treatment, an elevation of miR-19a and miR-19b levels was perceived that subsequently targeting BARD1 3’UTR expression enhancing the apoptotic activity of cancer cells [44].

Following a similar trend, BARD1 9’L, a specific alteration of the BARD1 gene, was defined to function as competing for endogenous RNA (ceRNA) that negatively modulates the expression of BARD1 mRNA as BARD1 9’L was reported to compete with miRNAs (such as miR-101 and miR-203) on their binding sites of BARD1 3’UTR [12]. On the other hand, the protein expression of BARD1 was found to be positively regulated by estrogen through activation of estrogen response element (ERE) on intron 9 of BARD1 [45]. The long non-coding RNAs (lncRNAs) display gene regulatory roles that modulate different biological mechanisms. GUARDIAN, a p53-responsive lncRNA, was determined to be involved efficiently in preserving genomic integrity and delivering protection against genotoxic stress. Furthermore, GUARDIAN, as an RNA scaffold, can enhance the heterodimerizing of BRCA1 with its partner interactor BARD1. Therefore, suppression of GUARDIAN resulted in disruption of BRCA1-BARD1 complex, ensuing genomic instability, induction of apoptosis, and augmented the adverse effect accompanied by genotoxic stress [46].

BRCA1and BARD1 convey solid contribution in controlling ATM/ATR pathway for DNA repair mechanism that is pertinent to cells fate decision. Histone modification of hESC was found to play a role in epigenetic modulation of the BARD1 gene. Regulation of alternative splicing process by H3K36me3 reduced BARD1 expression with subsequent suppression of ATM/ATR signaling that regulates hESC differentiation [47]. Another report revealed that in patients with hepatocellular carcinoma (HCC) that ended by the cirrhotic liver, the BARD1 gene showed significant hypomethylation (13.3%) in comparison to normal controls BARD1 hypomethylation was suggested as a predictive biomarker for predisposing to aggressive disease in HBV-negative patients [48].

#### Breast cancer mutations at a glance

Positive breast cancer family/personal history was considered a key player in hereditary predisposition to the disease occurrence. Around 30% of hereditary breast cancer cases result from mutations of rare but highly penetrant genes, including BRCA1, BRCA2, PTEN, TP53, CDH1, and STK11, which account for approximately 80% of breast cancer risk. Mutation in BRCA1 was first discovered in 1990 in families with reminiscent pedigree. Four years later, the variants in the BRCA2 gene were identified [49]. Mutations in BRCA1 or BRCA2 resulted in Hereditary Breast/Ovarian Cancer (HBOC) syndrome, yet some patients with this syndrome were negative for BRCA1 and BRCA2 alterations. Tumors with BRCA1/2 mutations are of a basal highly aggressive phenotype. Furthermore, mutations in rare but moderately penetrance genes that involve CHEK2, BRIP1, ATM, RAD50, RAD51C, MRE11, NBN, and PALB2 were also reported in 2–3% of the breast cancer cases. These genes were found to interact with BRCA1/2 and engaged in DNA repair mechanisms. A small number of SNPs was found to associate with common low penetrance alleles and increase the risk of breast cancer in a polygenic manner. These include RAD51D, BARD1, RAD51C, ABRAXAS, NBN, and XRCC2BRIP mutation. Clinically, patients with suggestive genetic predisposition were usually tested for mutations detected in the high penetrance gene group [50]. Moreover, a genome-wide association study (GWAS) of breast cancer declared identification of 65 novel loci associated remarkably with a high risk of breast cancer at *P* < 5 × 10^− 8^ such as FES, MAP 3 K11, CLK2, GRK7, USP25, DFFA, PKP1, and ZKSCAN3 [51].

#### Association of Cys557Ser BARD1 variant to breast cancer risk

The most reported mutation of BARD1 is a missense mutation of an amino acid substitution of cysteine with serine at position 557 (Cys557Ser) [52, 53]. A mutational analysis study was conducted among 126 Finnish breast and/or ovarian cancers families to investigate the potential contribution of BARD1 alterations to tumor development. The Cys557Ser missense variant was identified within the BARD1 region that was required to regulate apoptosis and transcriptional machinery and revealed higher frequency in the breast, but not ovarian cancer cases compared to healthy controls (7.4 vs 1.4%, *p* = 0.001). Interestingly, the index cases were negative for BRCA1 and BRCA2 mutations, highlighting that this mutation in familial predisposition to breast cancer is sufficient to cause the disease on its own [54]. Following this study, Stacey et al. and his colleague, in a computerized genealogy investigation examined the relationship between BARD1 Cys557Ser mutation and familial grouping of breast cancer using a cohort of 1090 Icelandic breast cancer patients with invasive type and 703 controls. Carriers of this variant revealed a higher risk of developing single and multiple primary breast cancer of lobular and medullary breast carcinomas than non-carriers. Furthermore, this risk increased up to 0.047 (OR 1/4 3.11, 95% CI 1.16–8.40, p 1/4 0.046) in the double carriers of both BARD1 Cys557Ser mutation and BRCA2 999del5 mutation [55]. Supporting previous reports, a case-control based analytic investigation was implemented among the Spanish/South American population. The selected participants showed a positive family history of breast cancer, yet they harbor intact BRCA1/2 genes with no aberrations. The C-terminal of BARD1 Cys557Ser was assessed and showed a significant increase in breast cancer probability (*P* = 0.04, OR = 3.4 [95% CI 1.2–10.2]). This probability was further increased in patients presented with double mutations of BARD1 Cys557Ser joint with XRCC3 241Met variant (*P* = 0.02, OR = 5.01 [95% CI 1.36–18.5]) among patients with a family history of breast or/an ovarian cancer [56].

On the other spectrum, an Australian study of two breast cancer case–control sample sets with positive breast cancer family history cases was conducted. In this population, the frequency of the BARD1 Cys557Ser variant was not significantly different in case-control cases (P0.3) and is not correlated with elevated breast cancer risk [57]. In agreement with these Australian findings, numerous other reports have failed to find a definitive association between BADR1 variant and breast cancer occurrence [58–60]. The role of BARD1 Cys557Ser varient or BARD1 haplotypes as modifiers of BRCA1/2 associated breast cancer risk was further evaluated in a cohort of 5546 BRCA1 and 2865 BRCA2 mutation carriers. No evidence of either BARD1 mutations supporting the significant association with breast cancer risk was obtained in both BRCA1 and BRCA2 mutation carriers with a total predicted effect of 0.90 and 0.87, respectively [61]. Furthermore, another group of researchers using DHPLC analysis of 210 breast cancer families (129 families have no mutations in BRCA1 or BRCA2) of Australian ethnicity have identified a set of nine coding mutations of BARD1 including two novel variants (Thr598Ile and Ile692Thr). Yet none of these nine mutations show to harbor a pathogenic impact based on their segregation, distribution, and frequency among the selected cases. Additionally, the three variants (1139del21, G1756C, and A2285G) that were associated with breast cancer in other populations were identified as non-pathogenic polymorphisms in their cases. Thus, BARD1 mutations/ polymorphism was not suggested as a high penetrance susceptibility gene in familial breast cancer evolution among the Australian community [60].

Collectively, while this BARD1 Cys557Ser mutation was reported to link to breast cancer incidence in Iceland, Finland, Spanish/South American, and Italy, other reports from Yoruba, Chinese, Japanese, Australian, and African-American individuals didn’t show similar findings [62, 63]. These inconsistent findings regarding the association of the BARD1 Cys557Ser variant to familial breast cancer susceptibility suggest that this mutation might be confined to the specific geographical substructure of the European population (due to regional migration) rather than de novo variant [55].

#### Influences of other BARD1 mutations on cancer predisposition risk

Numerous comprehensive sequencing studies have discovered many genetic alterations among different clinical samples [18, 64–66]. While the biological functions of BRCA1 have been well documented, the functional machinery of BARD1 hasn’t been fully addressed. In a report on hereditary breast and ovarian cancers, two BARD1 cis mutations, P24S and R378S, were identified. P24S mutation reduces the interaction affinity between BARD1 and BRCA1 while the R378S variant interferes with the nuclear translocation of the BRCA1/BARD2 complex. The simultaneous existence of these two mutations is found to contribute synergetically to tumor development in both in vitro and in vivo models. Additionally, these two variants are jointly disrupting DNA damage response impinging on genomic stability nevertheless neither of the individual mutation can produce damaging effects [67]. In a mutational study that included different types of gynecological cancers, ovarian, breast, and uterine tumors, seven polymorphisms were identified within the coding sequence of BARD1, including somatic missense mutations and germline alterations. These mutations were associated with loss of the wild type BARD1 allele resulting in tumors evolution and progression. BARD1 mutation (Gln564His) was detected in patients with the concurrent presentation of breast and endometrial carcinoma [63].

Furthermore, the Gln564His mutation of BARD1 was found to avoid p53-dependent apoptosis by decreasing binding to the polyadenylation cleavage specification complex (CSTF-50) [31, 34]. Therefore, studying BARD1 mutations, specifically in synchronous tumors presentation, was suggested.

A Chinese case- control study of 507 breast cancer cases and 539 matched controls showed the effect of three non-synonymous polymorphisms in the BARD1 gene (Pro24Ser, Arg378Ser, and Val507Met) was evaluated. These SNPs revealed low penetrance effects in the BARD1 gene on breast cancer predisposition with a remarkable reduction in breast cancer risk [68]. Contrary, another broad case-control designed study to examine the effect of nonsense mutation c.1690C > T (p.Q564X) was conducted among the European (Polish and Belarusian) population. A low/moderate increase in breast cancer risk associated with this nonsense variant was determined (OR = 2.30, *p* = 0.04) with further elevation in risk in the more aggressive breast cancer types including TNBCs, bilateral breast cancers, early-onset cancer, and familial breast and ovarian cancers [69]. In alignment with the European study, BARD1 mutation was recognized in 10,901 TNBC cases as one of the most common non-BRCA1/2 mutated genes that showed solid contribution to TNBC predisposition with an incidence of 0.5–0.7%. Additionally, African American carriers of BARD1 gene pathological variants were at higher risk of TNBC (39%) than Caucasian PVs carriers’ patients (21%) [39, 70].

A direct sequencing and SNaPshot analysis was used to identify the exon mutation of the BARD1 gene in 60 early-onset breast cancer cases and 240 healthy controls. A deletion mutation was identified at the rs28997575 site of BARD1 and showed an elevated risk of breast cancer by 3.4 times (*P* = 0.013) compared to the unaffected group. In contrast, another GC genotype missense variant at the rs2229571 site of BRDA1 was associated with a reduced risk of breast cancer by 72.6% (*P* = 0.001). Interestingly, most of the variants’ carriers have a strong family history of breast cancer compared to the control group. Thus, highlighting the substantial involvement of breast cancer positive family history increased the risk of genetic predisposition to breast cancer, particularly in BARD1 polymorphism carriers [71]. Likewise, in a large pooled analytical study of both breast cancer (48,000 cases) and ovarian cancer (20,800 cases), many pathogenic variants (PVs) of the BARD1 gene were cataloged. These PVs of BARD1 showed a moderate risk of breast cancer (odds ratio (OR) = 2.90, 95% CIs:2.25–3.75, *p* < 0.0001) but not ovarian cancer (OR = 1.36, 95% CIs:0.87–2.11, *p* = 0.1733). Thus, the BARD1 gene is a diagnostic biomarker in testing breast cancer patients [72].

Recently, three BARD1 inherited missense mutations were identified in the RING domain (Cys53Trp, Cys71Tyr, and Cys83Arg) in a family affected by breast cancer. The study showed that the mutant BARD1, with any of the mutations, could form a heterodimer with BRCA1; however, the mutant BARD1/BRCA1 complex could not bind to nucleosomes and cause loss of H2A ubiquitylation. These mutations also trigger a defect in transcriptional repression of the BRCA1-regulated estrogen metabolism genes CYP1A1 and CYP3A4, which are usually regulated by the H2A ubiquitylation pathway [73]. Also, a whole-exome sequencing study on 10,000 cancer samples from 33 cancer types has successfully identified 76 BARD1 cancer-associated missense and truncation variants. Remarkably, only two known benign mutations were associated with HDR, while four known pathogenic mutations did not have an association with HDR. BARD1 mutant cells showed higher sensitivity to DNA damage agents [74]. Indeed, a rare missense mutation of BARD1 gene c.403G > A or p.Asp135Asn was detected in TNBC patients. This mutation was found to enhance the response of breast cancer cells to PARPi therapy [75]. Although there are more abundant BARD1 isoforms that are widely expressed in different types of cancer, their real pathogenic effect is due to alternative splicing and expression of the oncogenic dominant-negative form [11, 76],

Aside from breast cancer, BARD1 gene polymorphism was also demonstrated in neuroblastoma (NB) cases. Three BARD1 gene polymorphisms (rs7585356 GNA, rs6435862 TNG, and rs3768716 ANG) were highly associated with neuroblastoma risk. Using the TaqMan approach applied to 145 cases and 531 controls, only the rs7585356 GNA polymorphism revealed remarkable results in association with an increased susceptibility to nephroblastoma (odds ratio (OR) = 1.78, 95% confidence interval (CI) = 1.01–3.12] with stage I + II clinically [77]. A Chinese report has investigated the risk of eleven BARD1 SNPs in NB development. Seven out of eleven BARD1 SNPs revealed increased risk of high stage (III/IV) NB occurrence these include one SNP in 5′-UTR (rs17489363 G > A), two SNPs in exon (rs2229571 G > C and rs3738888 C > T), and four SNPs in an intron (rs3768716 A > G, rs6435862 T > G, rs3768707 C > T and rs17487792 C > T) [78]. The variant (rs17489363 G > A) was reported to be the most frequent SNPs in the BARD1 gene correlated to NB and associated with a reduction in the transcription of FL- BARD1 [48].

A common mutation at site c.1361C > T results in the skipping of exon 5, which disrupts ANK repeat domains, binding part of the splicing factor SC35 that affects apoptosis in the ovarian cancer cell line NuTu-19 [79]. The NuTu-19 cell line was resistant to the induction of apoptosis; however, it became sensitive to apoptosis after exogenous expression of full-length gene BARD1, suggesting that the absence of exon 5 leads to abnormal isoforms that have lost their tumor-suppressor potential, affecting the apoptosis pathway [80]. In addition, other studies have reported different BARD1 mutations linked to various gynecological cancers such as fallopian tubes, ovarian, and cervical cancers involving c.1977A > G, p.Gln715Ter, c.2148delCA, and p.Thr716fs*12 [80–82].

Together, these findings from accumulating evidence have concluded a high/moderate risk of breast (or others) cancers harnessed to various BARD1 polymorphisms in a context dependant manner. Hence subsequent practical and experimental investigations are in need to further validate the above data.

## The scope of the paper and methodologies

In this work, we focused on exemplifying the expression profile of BARD1 on both mRNA gene expression and protein levels in breast cancer samples and constructing a correlation between BARD1 expression level and different clinico-pathological features as well as patients’ prognosis. Identifying the interactors partners proteins with Bard1 and possible mechanisms involved in breast cancer tumorigenesis. Elucidating the expression and dysregulation of BARD1 in other types of human cancers. Finally, deliberating the value of using the FL- BARD1 and BARD1 isoform as a prognostic and therapeutic target in cancer treatment. Herin, we attempted to take the advanteges of using various computational methodological approaches and powerfull bioinformatics tools to construct our study. This approach is well recognized [83–85] and provides decent insight and understanding of the biological role of BARD1 in breast cancer evolution.

### Assessment of the effect of BARD1 gene on survival by Kaplan-Meier plots (KM-plot)

Kaplan-Meier plots were analyzed using online KM plotter software (http://kmplot.com/analysis/) [86]. The tool analyses the effect of 54,675 genes on the survival outcome of patients using 10,293 cancer samples from the Affymetrix microarray data in the Gene Expression Omnibus (GEO: http://www.ncbi.nlm.nih.gov/geo/), the European Genome phenome Archive (EGA: https://ega.crg.eu/) and The Cancer Genome Atlas (TCGA: http://cancergenome.nih.gov/) databases. We analyzed the potential effect of BARD1 gene expression on different parameters including overall survival (OS), distant metastases free survival (DMFS), and relapse free survival (RFS). In a large cohort of cancer patients, including patients with gastric (1065), ovarian (1816), lung (2437), and breast (5143) cancers. The cohort of the patients was split by using an auto select best cut off option. The selected follow up threshold is 10 years (120 months) in all analyzed parameters (OS, RFS, and DMFS). The hazard ratio (HR) with 95% confidence intervals and log rank *P*-value (below 0.05 were considered significant) were calculated. Biased arrays were excluded for quality control. The BARD1 and BLM genes expression signature was obtained using the multigene classifier option and the mean expression levels of both genes tool provided by the KM plotter. The Affymetrix IDs that are used for BARD1, and BLM are 205345_at and 205733_at respectively.

### Protein-protein interaction network (STRING)

We used the Search Tool for the Retrieval of Interacting Genes/Proteins database (STRING v10.5) (https://string-db.org/) [87] to construct the PPI network associated with BARD1 protein. This tool provides information about both the physical and functional interaction of the predicted proteins. When given a list of the proteins as input, STRING can search for their binding partners and generate a PPI (protein-protein interaction) network, which is a network of all the interactions between the inputted proteins and their binding partners. First, based on the inputted seed proteins, we constructed the PPI network associated with HUD obtaining the seed proteins and their neighbors. The interactions were derived from high-throughput lab experiments and previous knowledge in curated databases at the high level of confidence (sources: experiments, databases; score ≥ 0.90). Next, we used the string system to simplify the network between BARD1 and other genes involved in cancer development and progression.

### Data mining of GOBO

Is Gene expression-based Outcome for Breast cancer (GOBO) online database (http://co.bmc.lu.se/gobo/) [88] that includes 1881 breast cancer patients. This online analysis tool was used to graph the correlation between the gene co-expression of BARD1 and BLM genes and the breast cancer molecular subtypes (PAM50), tumor grades, and ER status of the breast cancer.

### Breast Cancer gene-expression MINER (MINER)

The Breast Cancer Gene-Expression Miner v4.8 (bcGenExMiner v4.8) (http://bcgenex.ico.unicancer.fr/BC-GEM/GEM-Accueil.php?js=1) [89] is an online dataset of 36 published genomics data (about 5696 patients). We utilized this tool to examine the expression of BARD1 gene (selecting targeted analysis) in relation to different clinico-pathological features including tumors grades, stages, lymph nodes status, and the expression of the classic biomarkers (ER, PR, HER2, and TP53) that used in stratification of breast cancer subtypes. Also, by using correlation module targeted analysis of this database we map a correlation between Bard1 protein and its partners interactors' proteins.

### The human protein atlas

The Human Protein Atlas allows us visualing the map of all human protein-coding genes expression patterns in different cells, tissues, and organs both in normal and across the 20 most common types of cancer (www.proteinatlas.org) [90]. This atlas program uses various methods to catalogue the data including antibody-based technique, mass spectrometry-based proteomics, transcriptomics, and systems biology. We used the pathology/cancer section of this database to picture the expression level of BARD1 in different cancers especially breast cancer patients’ samples. This section provides data based on mRNA and protein expression from 17 different types of human cancer, immunohistochemistry images analysis, and KM-Plot prognostic data tool.

## Results and discussion

### Expression level of BARD1 in breast cancer patients

**Fig. 2:**
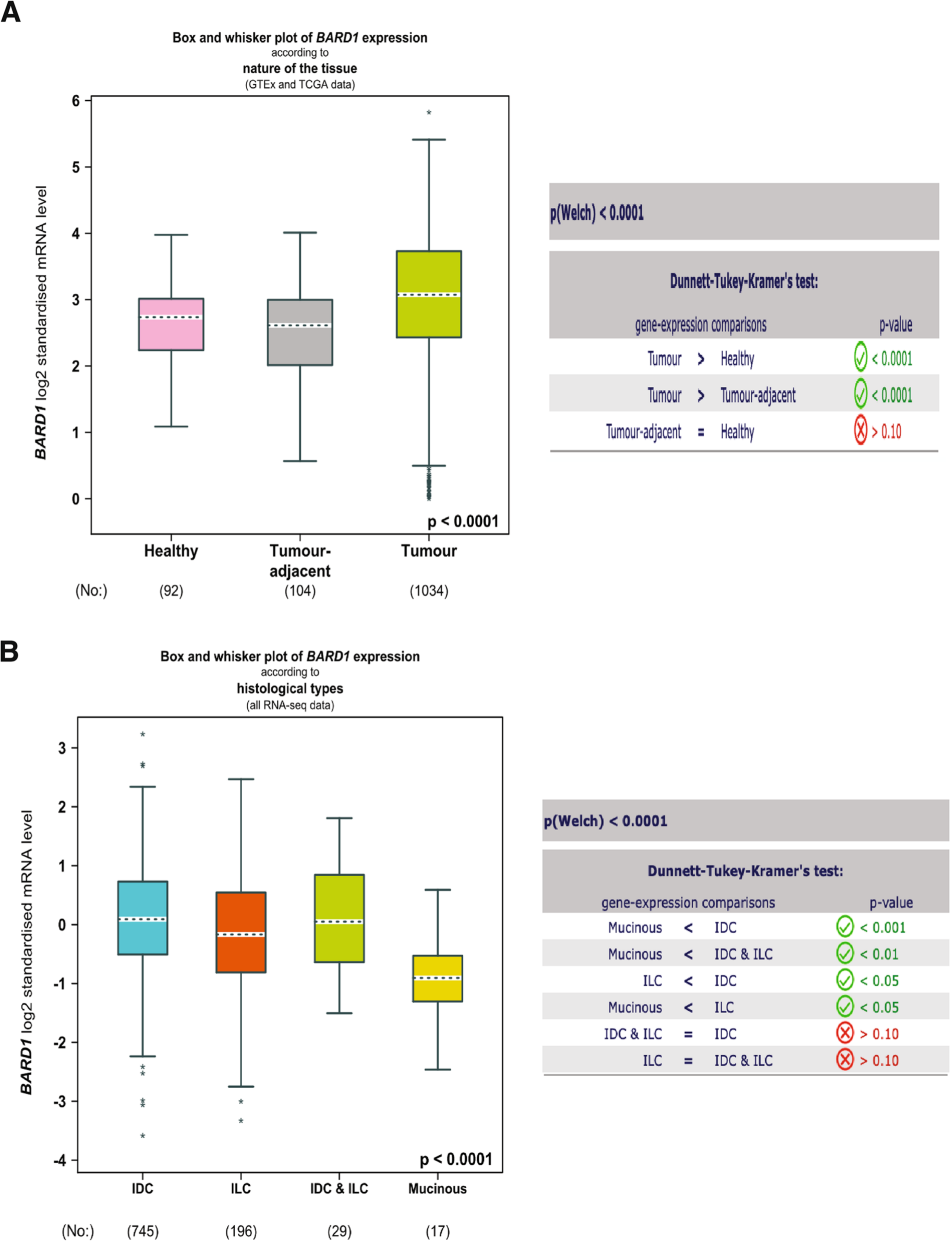
Assessment of BARD1 mRNA expression profile in breast cancer patient: **A** BARD1 mRNA expression level according to the nature of the tissues in healty (92), tumor adjacent (104), and tuomrs tissues (1034), left panel. Table represents the caculated *P*-value in gene expression comparsion (right panel). **B** BARD1 mRNA expression level according to hitological types, IDC (745), ILC (196), IDC & ILC (29), Mucinous (17), left panel. Table represents the caculated P-value in gene expression comparsion (right panel). IDC: invasive ductal carcinomas, ILC: invasive lobular carcinomas. Source: Database of The Breast Cancer Gene-Expression Miner v4.8 (bcGenExMiner v4.8) (http://bcgenex.ico.unicancer.fr/BC-GEM/GEM-Accueil.php?js=1) [61]

**Fig. 3:**
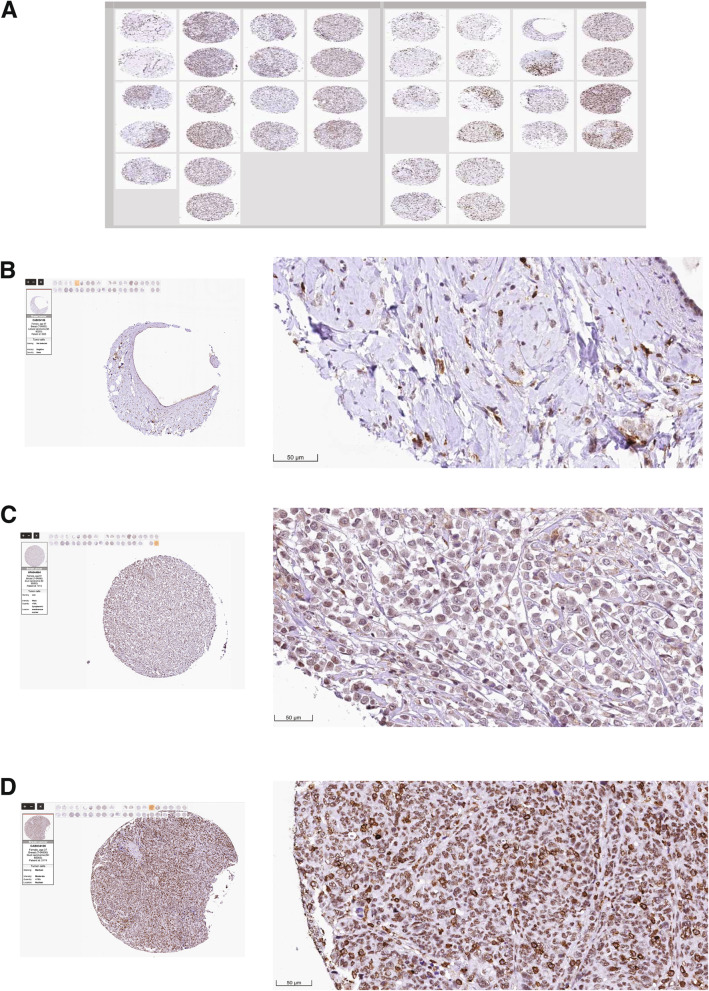
Visualization of BARD1 protein expression profile in breast cancer patients: **A** Immunohistochemical analysis of BARD1 expression in 38 breast cancer patients’ samples using HPA044864-Sigma antibody against BARD1 protein. **B** Negative/ Undetectable staining. **C** Low intensity nuclear staining of BARD1. **D** Medium intensity nuclear staining of BARD1. Scale bar is 50 μm. Source: THE HUMAN PROTEIN ATLAS online tool (www.proteinatlas.org) [62], https://www.proteinatlas.org/ENSG00000138376-BARD1/pathology/breast+cancer#

To better understand the role of BARD1 in breast cancer progression, we investigated the gene expression level of BARD1 in a large cohort of 5696 breast cancer patients using a publically available database of The Breast Cancer Gene-Expression Miner v4.8 (bcGenExMiner v4.8) [89]. As shown in Fig. 2A, while there is no difference in BARD1 expression level between the healthy and the tumor adjacent breast tissues (***P*** = 0.10), we found a significant elevation in BARD1 expression in breast cancer tumor tissues in comparsion to both healthy and tumor adjacent tissues (***P*** < 0.0001). To further examine its involvement in breast cancer tumorogenesis, we used the same database to analyze BARD1 expression in association with different histological classes. BARD1 gene expression showed the highest level in invasive ductal carcinomas (IDC) in comparison to other classifications, invasive lobular carcinomas (ILC, ***P*** < 0.05) and mucinous carcinomas (***P*** **< **0.001) (Fig. 2B). IDC is the most common breast cancer presenting type (~ 75–80%) that is characterized by invasion to surrounding tissues and lymphatic systems, and by distant metastasis [91, 92]. To validate the expression profile of BARD1 in breast cancer tissues, next we utilized THE HUMAN PROTEIN ATLAS online tool (www.proteinatlas.org) [90], that provides information about the expression level of the protein coding genes from 20 different types of the common human cancers. Immunohistochemistry (IHC) analysis of BARD1 protein levels in 38 breast cancer patients samples (Fig.3A) revealed undetectable/negative, low to medium staining of BARD1 protein levels in different breast cancer histological types (Fig. 3B, C, D). Indeed, the intensity of staining is usually correlated positively with the aggressivity of cancer and the size of the tuomrs [13, 93]. Together these data indicate that BARD1expression is upregulated during breast cancer evolution thus supporting a potential an oncogenic role of BARD1 in breast cancer patients.

### BARD1 gene expression level in association with clinicopathological characters in breast cancer patients

**Fig. 4:**
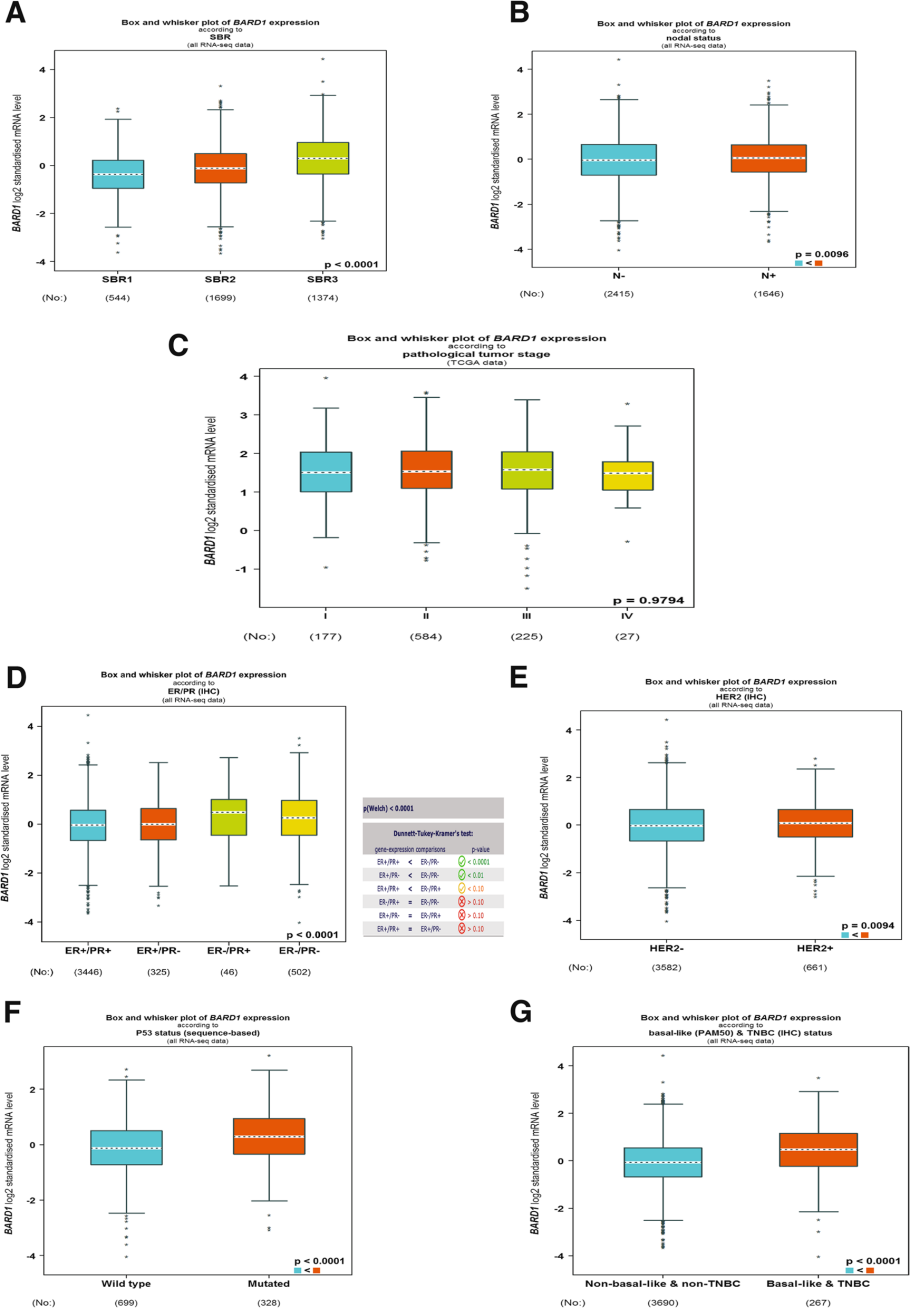
Evaluation of BARD1 gene expression level in relation with clinicopathological characters in breast cancer patients: **A** BARD1 mRNA expression level according to histological Scarff Bloom & Richardson grade status of breast cancer, SRB1(544), SRB2(1699), SRB3(1374). **B** BARD1 mRNA expression level according to lymph node (N) status in breast tuomrs, N- (2415) and N+ (1646). **C** BARD1 mRNA expression level in correlation with clinical stages, I (177), II (584), III (225), IV (27). **D** BARD1 mRNA expression level in association with ER/PR expression, ER+/PR+ (3446), ER+/PR- (325), ER−/PR+ (46), ER−/PR- (502). **E** BARD1 mRNA expression level according to HER2 enriched, HER2- (3582) and HER2+ (651). **F** BARD1 mRNA expression level according to TP53 mutation status, wild type (699) and mutated (328). **G** BARD1 mRNA expression level in non-basal & non-TNBC (3690) in comparison to basal & TNBC tumor tissues (267). Source: Database of The Breast Cancer Gene-Expression Miner v4.8 (bcGenExMiner v4.8) (http://bcgenex.ico.unicancer.fr/BC-GEM/GEM-Accueil.php?js=1) [61]

To further examine the contribution of BARD1 in breast cancer tumorogenesis, next we analyzed the gene expression level of BARD1 in association with different clinicopathological parameters such as tumors grades and stages [94, 95] using the same bioinformatics dataset of bcGenExMiner v4.8 [89]. Our results (Fig. 4A) showed a significant upregulation of BARD1 mRNA level in a higher grade (Gtrade3, SBR3” Scarff Bloom & Richardson grade status**”**), which is a poorly differentiated tumors featured with faster growing and higher tendency to metastasize in comparison to other grades (Grade1(SBR1) and Grade2(SBR2)) (***P*** < 0.00001) [94, 95]. Moreover, using the same analytical tool, a higher BARD1 mRNA level was found to be significantly (***P*** **= **0.0096) associated with lymph node positive breast cancer patients (LN+), as revealed by Fig. 4B. In contrast, no differences (***P*** = 0.9794**)** were observed in BARD1 mRNA levels between different breast cancer stages (Stage I – Stage IV), (Fig. 4C). Next, we evaluated the expression of BARD1 mRNA in relation to ER, PR, HER2, and TP53 status, the classic biomarkers that are used to stratify breast cancer into different molecular subtypes [96]. Indeed, our findings (Fig. 4D) showed that elevated expression of BARD1 mRNA levels is associated with ER−/PR- tumors (***P*** **< **0.0001). On the other hand, BARD1 mRNA level was observed to be upregulated in HER2 enriched breast cancers in comparison to HER2 negative tumors (Fig. 4E), ***P*** = 0.0094. Importantly, we found a significant correlation, ***P*** < 0.0001, between higher BARD1 mRNA level and TP53 mutated breast cancer tumors (Fig. 4F). TP53 harbors an onco-suppressive function and TP53 mutation is considered a driving factor in the development of triple negative breast cancer, TNBC, the most breast cancer aggressive phenotype [97]. Therefore, next we examined the expression of BARD1 mRNA in TNBC/Basal tumors. As shown in Fig. 4G, BARD1 mRNA level demonstrated significant elevation, ***P*** < 0.0001, in Basal and TNBC breast cancer samples. These findings are in alignment with a previous report that identified the utmost expression level of BARD1 in the Basal and HER2+ breast cancer molecular subtypes [98]. Taken together, these results suggest that BARD1 expression is correlated with poorly differentiated aggressive breast cancer phenotype.

### Higher BARD1 gene expression level is associated with poor patient outcome in breast cancer

**Fig. 5:**
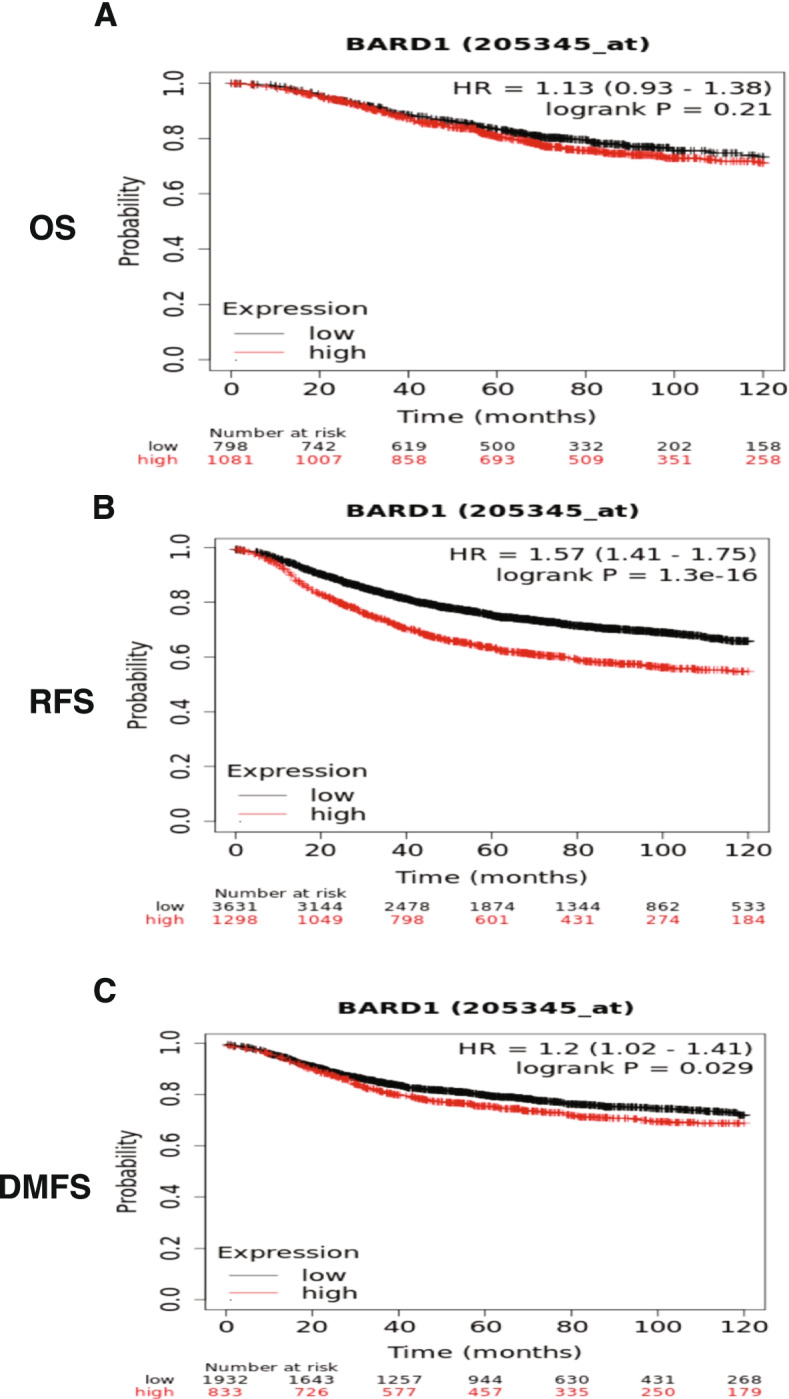
The Kaplan Meier plotter shows the survival curve in breast cancer patients: **A** No significant correlation between the high expression of BARD1 gene and the overall survival (OS), *P* = 0.21. **B** & **C** Represent the high expression of BARD1 gene is significantly associated with shorter relapse free survival (RFS) and distant metastases free survival (DMFS), *P* = 1.3^-16 and *P* = 0.029, respectively. Source: The Kaplan-Meier Plotter, (https://kmplot.com/analysis/) [71]

The above results demonstrae the decisive engagement of BARD1 in the breast cancer development and progression. To complete the picture, we verified the prognostic value of BARD1 expression in the breast cancer patient outcomes. For this aim, we used the Kaplan-Meier (KM) plotter, a gene profiling tool that includes data of 5143 breast cancer cases in relation to several prognostic parameters, overall survival (OS), relapse free survival (RFS), and distant metastasis free survival (DMFS) (https://kmplot.com/analysis/) [99]. While we did not find a significant effect of high BARD1 gene expression levels on the overall survival of breast cancer patients (***P*** = 0.21), interestingly, patients with elevated BARD1 gene expression levels displayed significant shorter RFS (***P*** = 1.3^-16) and DMFS (***P*** = 0.029) and consequently unfavorable prognosis (Fig. 5A, B, and C).

### BARD1 interaction network with parteners proteins

**Fig. 6:**
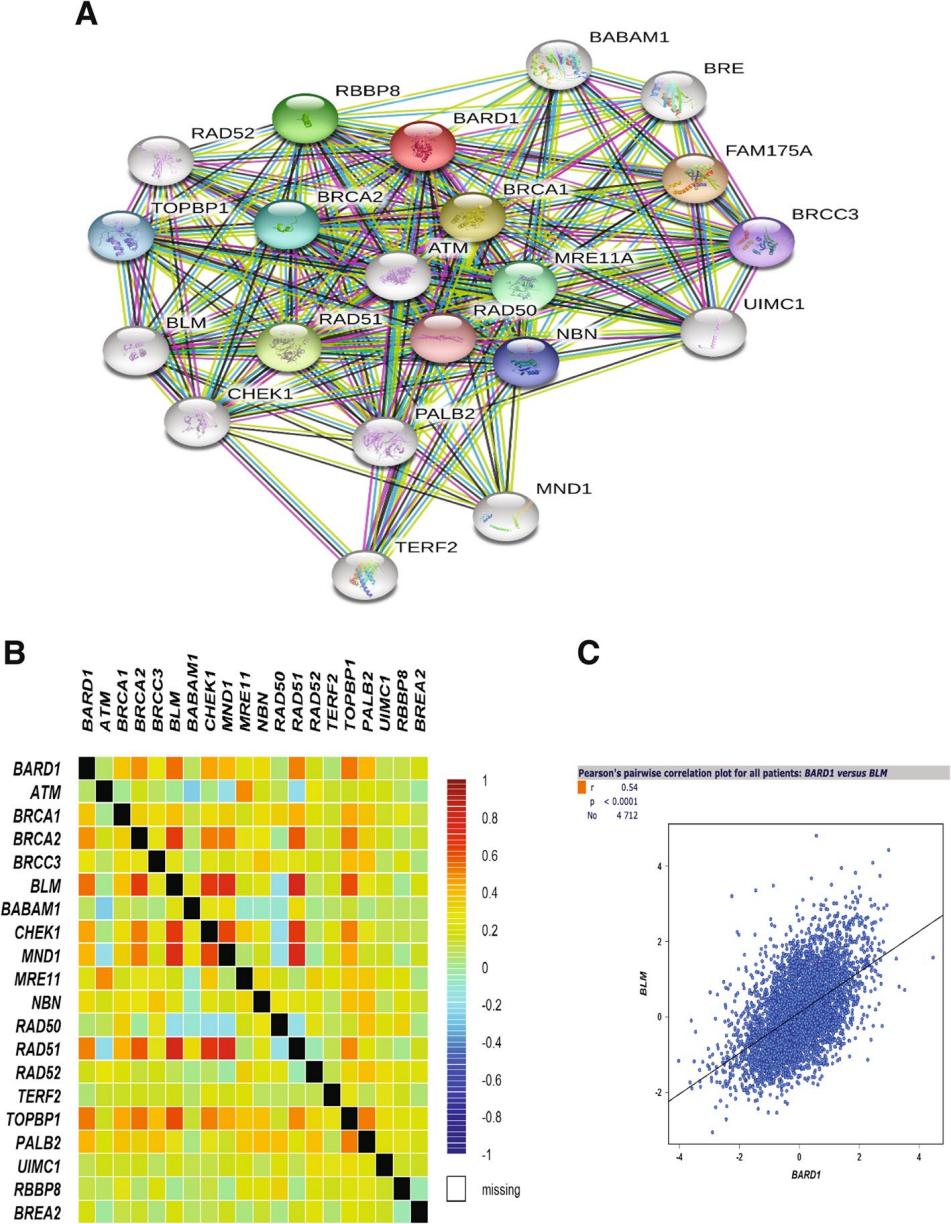
Protein-Protein interaction network: **A** Diagram showing the network of the BARD1 interaction pattern with partners proteins generated by STRING. **B** The BARD1-Proteins interaction map showing different degrees of correlation strength by bcGenExMiner. **C** Pearson’s pairwise correlation graph shows high degree of positively correlated interaction between BARD1 and BLM genes, r = 0.54 and *P* < 0.0001 in breast cancer patients (4712), by bcGenExMiner. Source: Retrieval of Interacting Genes/Proteins database (STRING v10.5) (https://string-db.org/) [59]. Database of The Breast Cancer Gene-Expression Miner v4.8 (bcGenExMiner v4.8) (http://bcgenex.ico.unicancer.fr/BC-GEM/GEM-Accueil.php?js=1) [61]

To identify the likely mechanistic pathway by which BARD1 exhibited its tumorigenic effect in breast cancer, we scrutinized the interaction between BARD1 and other proteins that might also involve in the processes of carcinogenesis. For this aim, we used the Search Tool for the Retrieval of Interacting Genes/Proteins database (STRING v10.5) [87] to construct the protein-protein interaction (PPI) network associated with BARD1 protein. As shown in Fig. 6A, 20 anticipated partners of BARD1 were retrieved in the network at the protein level, this finding is in agreement with previous reports [1, 98]. Numerous protein interactors have been identified to be involved in DNA repair machinery such as MRE11A, RAD50, RAD51, and PALB2 [1]. Other genes, like ATM, CHEK2, NBN, and RAD50 were reported to contribute to breast cancer progression in association with BARD1 [93, 100]. Next, we conducted a comperhensive analysis to map the correlation between BARD1 and the interactors proteins using bcGenExMiner v4.8 analytical tool [89] (Fig. 6B). Either equivalent or positively correlated interactions were obtained between BARD1 and the interactor proteins with the highest Pearson’s pairwise correlation was perceived with BLM protein (r = 0.54, ***P*** **< 0.0001**), (Fig. 6C).

### BARD1/BLM co-expression is associated with poor patient outcomes

**Fig. 7:**
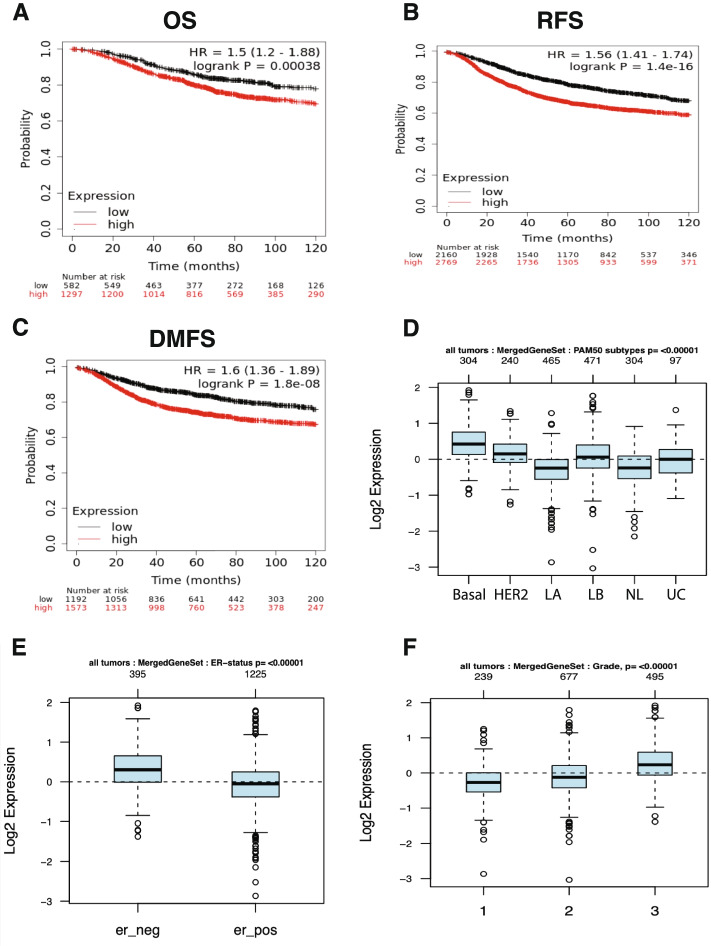
BARD1/BLM co-expression correlated with worse patient outcomes and aggressive breast cancer phenotype: **A**, **B** & **C** Represent the high expression of BARD1 gene is significantly associated with worse OS (*P* = 0.00038), RFS (*P* = 1.4^-16), and DMFS (*P* = 1.8^-08), by KM-Plot. **D** BARD1-BLM gene signuture expression level according to breast cancer molecular subtypes, basal (304), HER2 + (240), Luminal A, LA (465), Luminal B, LB (471), Normal like, NL (304), and Unclassified, UC (97). **E** BARD1-BLM gene signuture expression level according to ER status, ER- (395) and ER+ (1225). **F** BARD1-BLM gene signuture expression level in correlation with histological grades, G1 (239), G2 (677), and G3 (495), by GOBO. Source: The Kaplan-Meier Plotter, (https://kmplot.com/analysis/) [71]. Gene expression-based Outcome for Breast cancer (GOBO) online database (http://co.bmc.lu.se/gobo/) [60]

BLM is a nuclear helicase protein that plays an essential role in maintaining genomic stability and DNA integrity. We reported earlier on the elevation in BLM mRNA expression in several types of cancers and that dysregulation of BLM is correlated with poor prognosis [15]. While we didn’t find previously a significant correlation between high BLM mRNA expression and breast cancer patients outcomes [15] herein, interestingly, we found that higher mRNA co-expression levels of both BARD1 and BLM are significantly correlated with poor OS (***P*** = 0.00038), RFS (***P*** = 1.4^-16), and DMFS (***P*** = 1.8^-08) in breast cancer patients, using KM plotter database calculating the mean expression values of both BARD1 and BLM genes (Fig. 7A, B, and C). To further elucidate the prognostic power of these genes' signature in relation to different breast cancer molecular subtypes, ER status, and tumor grade/differentiation condition, we used the Gene expression-based Outcome for Breast cancer Online (GOBO) dataset that includes information about 1881 breast cancer patients [88] to generate a gene set composed of BARD1 and BLM. As can be seen in Fig. 7D, E, and F our results disclosed a significant association between higher expression of BARD1-BLM genes signature and the basal breast cancer subtype (***P*** < 0.00001), ER negative tumors (***P*** < 0.00001), and higher tumor grade (Grade 3, ***P*** < 0.00001). This data should shed the light on the synergetic oncogenic role of the concurrent expression of BARD1 and BLM genes in breast cancer patients and thus expression is coupled with higher malignancy degree and wors patients' prognosis.

### BARD1 as a prognostic marker in other types of cancers

**Fig. 8:**
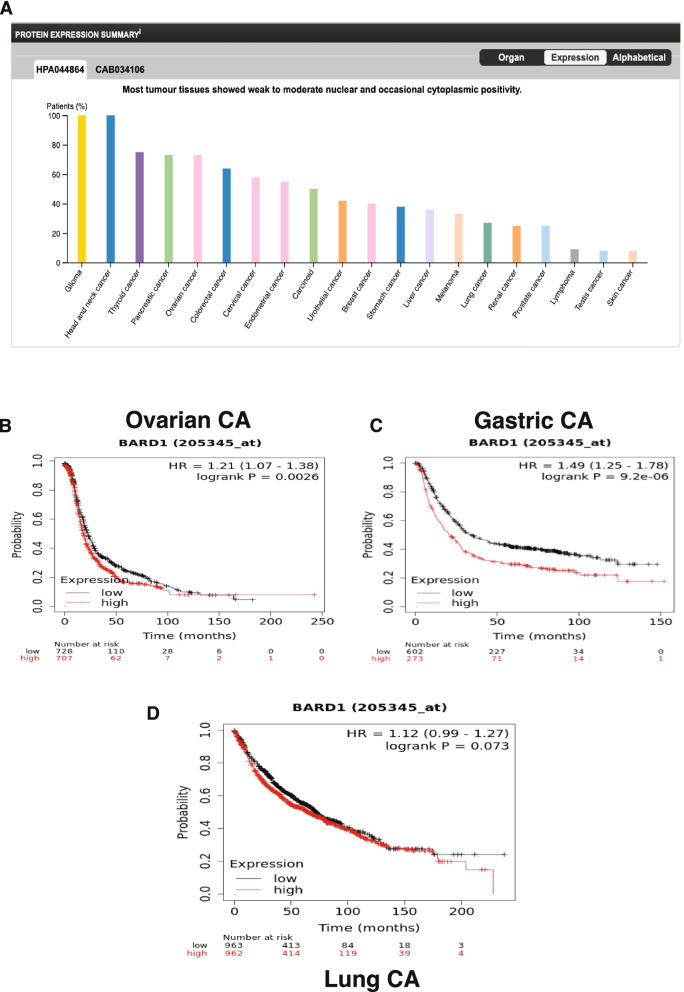
BARD1 as a prognostic marker in other types of cancers: **A** BARD1 protein expression summary in different human cancers, by the human protein atlas tool. **B** and **C** Represent the high expression of BARD1 gene is significantly correlated with poor OS in ovarian cancer (*P* = 0.0026) and gastric cancer (*P* = 2.9^-06), by KM-Plot. **D** No significant correlation between the high expression of BARD1 gene and the OS in lung cancer (*P* = 0.07). Source: THE HUMAN PROTEIN ATLAS online tool (www.proteinatlas.org) [62], https://www.proteinatlas.org/ENSG00000138376-BARD1/pathology. The Kaplan-Meier Plotter, (https://kmplot.com/analysis/) [71]

BARD1 was reported to be broadly expressed in many types of mammalian tissues both normal and cancers [101]. To explore the Bard1 protein levels in different types of human cancers, we used THE HUMAN PROTEIN ATLAS online tool. As can be seen in Fig. 8A, immunohistochemistry analysis of 20 different human cancers showed varient BARD1 protein expression levels, with the highest protein level was found in gliomas and head and neck cancers while the lowest levels were detected in testicular and skin cancers. Next, an online Kaplan-Meier plot was used to assess the prognostic effect of the BARD1 gene expression level on OS in breast (*n* = 5143), ovarian (*n* = 1816), lung (*n* = 2437), and gastric (*n* = 1065) cancers. We found a significant association between low expression of BARD1 gene in ovarian cancer (***P*** **= 0.0026**) and gastric cancer (***P*** **= 2.9^-06**) and better overall survival (Fig. 8B, C, and D). On the otherhand we didn’t find a significant association between BARD1 gene expression level and OS of lung cancer patients (***P*** **= 0.07**).

In 2012, Zhang and colleagues [7] reported different BARD1 isoforms in the colorectal tissues of 168 colon cancer patients, which were identified by immunohistochemical staining using specific antibodies and mRNA expression level analysis. The BARD1 isoforms identified include N19, PVC, WFS, and C20 to recognize exon 1 (N-terminus), the end of exon 3 (after RING region), and the beginning of exon 4, and exon 11 (C-terminus), respectively, in colon tissues [7, 11, 24, 36]. Their studies suggested that the loss of BARD1 expression (full-length) and expression of at least one BARD1 isoform could be essential for tumor growth. Consequently, they concluded that BARD1 isoforms protein products could affect the localization of BRCA1, and those isoforms might have oncogenic functions [8, 11, 76]. In addition, they determined that the oncogenic isoform overexpression is consistent with a lack of BARD1 promoter methylation, which was observed in all colorectal cancer samples. A similar observation was reported in ovarian cancer cells that lack promoter methylation of the BARD1 gene. Moreover, overexpression of BARD1 isoform mRNA and N19-positive staining were determined to be significantly associated with females with colorectal cancer [8, 11, 76].

In colon tumor tissues specifically, the expression of BARD1 isoforms κ, β, and π was significantly linked with tumorigenesis and invasiveness, while isoforms γ and φ might have an inhibitory effect. Therefore, the authors concluded that the lack of full-length BARD1 is a negative prognostic marker, but BARD1 isoforms could be used as prognostic markers for response to treatment regimens in colon cancer [7, 42]. Another study by Ozden et.al has also shown that BARD1β is upregulated in colon cancer cells [43]. This in turn triggers a more malignant phenotype and diminished RAD51 foci formation. Further, upregulated BARD1β caused a decrease in BRCA1 E3 ubiquitin ligase activity and its nuclear localization. This leads to less efficient or defective HR in colon cancer cells with BARD1β overexpression. Remarkably, BARD1β sensitized the colon cancer cells to the PARP1 inhibitor even in the presence of a wild-type BRCA1. Therefore, the authors suggested the potential use of BARD1β expression in colon cancer as a biomarker for treating patients with advanced colon cancer with the PARP1 inhibitor targeting the HR pathway [43].

Li et al. evaluated different oncogenic BARD1 isoforms that are expressed in gynecological cancers including ovarian, breast and cervical cancer cell lines (in vitro) and in situ tissue samples from a total of 106 cases of ovarian cancer, including serous, endometroid, mucinous and clear cell carcinoma at different tumor stages. They recorded the complete loss of full-length BARD1, or a decreased abundance compared to other isoforms, in all cancer cell lines derived from potentially hormone-controlled gynecologic cancers. The most common prognostic markers that were detected were isoforms Ω1 or Ω2 in ovarian, breast, and endometrial cancer cell lines with a lack of expression of full-length BARD1 in all collected samples [11].

The expression of epitope WFS (on isoforms π, κ, and β) and/or the PVC epitope (on isoform π only) was significantly correlated with decreased patient survival in patients with non-small cell lung cancer tumors (NSCLC). These results were confirmed in a chemically induced lung cancer mouse model. Specific immunostaining of epitopes PVC and WFS showed their overexpression in invasive, but not in confined, lung tumors in the mouse model. It was suggested that isoforms of BARD1 might be involved in different tumor stages, including initiation and invasive progression, which in turn might represent a new NSCLC prognostic marker [7, 79].

### Cancer screening and treatment via BARD1 isoform repression

A better understanding of genetics is the key to advancing the oncology field of both arms: screening/diagnosis and treatment. Recently, several studies have designed different epigenetic modulating compounds that are used in cancer treatment or preclinical development and are approved by the FDA [102–104]. Histone deacetylase inhibitors (HDACi) represent the most common epigenetic compounds that have efficacy against hematological malignancies and solid cancers and can affect different cellular mechanisms involved in the oncogenic properties of cancer cells [105, 106]. Examples of these compounds are suberoylanilide hydroxamic acid (Vorinostat, SAHA) [107], class I-specific HDACi Entinostat (MS-275) [108], class-specific modulators (class II inhibitor MC 1568) [109], and the HDAC6 inhibitor ST-80 [110]. Nuclear HDAC stabilizes DNA-histone complexes by removing the acetyl group of the histone N-terminal end, which increases its positivity and in turn increases electrostatic interactions with DNA thus repressing the transcription process [111–113].

Lepore et al. determined that Vorinostat (HDACi) decreases the mRNA levels of BARD1 by increasing the expression of miR-19a and miR-19b microRNAs [105]. MicroRNAs (miRNAs) are small noncoding RNAs that recognize a complementary sequence of a specific mRNA within the 3’untranslated region (3’UTR). miRNAs bind to the complementary sequence and can induce degradation or block translation of the targets through the complete/incomplete match of the miRNA-mRNA complex [114]. miR-19a and miR-19b were found to target BARD1 and belong to the miR-17-92 cluster known as ‘oncomir-1’. They are amplified in lymphomas and different solid tumors including lung, breast, and colon cancers [115]. It was hypothesized that lower levels of miR-19a/b coupled with BARD1 oncogenic isoforms overexpression can promote cancer advancement and vice versa. Vorinostat (HDACi) treatment increases miR-19a/b, which in turn leads to caspase-9 hyperactivation and targets Bim mitochondrial protein, which activates the intrinsic pathway of cellular apoptosis [116, 117].

Full length BARD1 has a tumor suppressor function while BARD1 isoforms can antagonize this effect and lead to oncogenesis [39]. These oncogenic isoforms can be produced excessively by breast cancer cells that can lead to an acceleration of breast cancer progression [12]. Also, the overexpression of BARD1 oncogenic isoforms was found to be involved in many cancers such as ovarian, colon, hepatic, gastric, and lung cancers [39]. Providentially, there are many antibodies have been developed to examine the expression of these isoforms [118, 119]. Moreover, a study found that BARD1 gene germline mutation is associated with poor patient outcomes and early development of breast cancer [120]. Likewise accumulating evidence supports the association between the expression of different BARD1 mutation and breast cancer pathogenesis [39, 48, 63, 67, 68, 70, 77, 78]. Bearing this in mind, it seems that screening of the mutated BARD1gene carriers and using the available antibodies to detect the oncogenic BARD1 isoforms would be beneficial in choosing the appropriate cancer therapy and expanding the oncological field.

Furthermore, about 70% of breast cancer patients are positive for the expression of estrogen receptor (ER), in which the mainstay of treatment is the endocrine therapy such as tamoxifen and aromatase inhibitors [121], which suppresses estrogen production [122]. Up to 25% of the ER+ breast cancer cases developed resistance to tamoxifen, either innate or acquired resistance [123, 124]. Recently, a study conducted on tamoxifen-resistant breast cancer cells found that both BARD1 and BRCA1 are overexpressed in these cells, and this led to resistance to DNA damaging chemotherapeutic agents such as Cisplatin and Adriamycin. Intriguingly, knocking down the expression of BARD1 and BRCA1 in these cells or inhibiting BRCA1 phosphorylation resulted in regaining sensitivity towards Cisplatin [65, 124, 125]. Additionally, it was found that upregulated BARD1 and BRCA1 are caused by activation of the PI3K/AKT pathway (an intercellular signaling pathway that is associated with cell cycle regulation) [126]. Inhibition of this pathway resulted in downregulation of BARD1 and BRCA1 in tamoxifen-resistant breast cancer cells and increased their sensitivity to Cisplatin. Thus, the study suggested that PI3K inhibitors can be used to re-sensitize ER-positive breast cancer patients to chemotherapy and radiotherapy [65, 124, 125]. On the other hand, analysis of TNBC core biopsy specimens of patients who received a full course of neoadjuvant chemotherapy showed hypermethylation of the BARD1 gene. However, the expression of this BARD1 hypermethylation was not significantly influenced the sensitivity to chemotherapy in TNBC specimens [127].

Breast and ovarian cancers presented with BRCA1/2 mutations exhibit therapeutic sensitivity and clinical response to PARPi chemotherapy. USP15 (deubiquitylating enzyme) implies an essential role in modulating the sensitivity of cancer cells to PARPi by regulating the HR mechanism. Indeed, USP15 through interaction with DSBs stabilize the BARD1 BRCT domain resulting in BARD1-HP1γ interaction and subsequent promotion of BRCA1/BARD1 complex recruitment at DSBs. Therefore, mutation or reduced expression of USP15 disturbes the genomic integrity, unbound USP15-BARD1 interaction, and further enhances the response to PARPi [128]. Thus, BARD1 expression/interaction can affect the response to various cytotoxic agents in a context dependent manner.

**Fig. 9:**
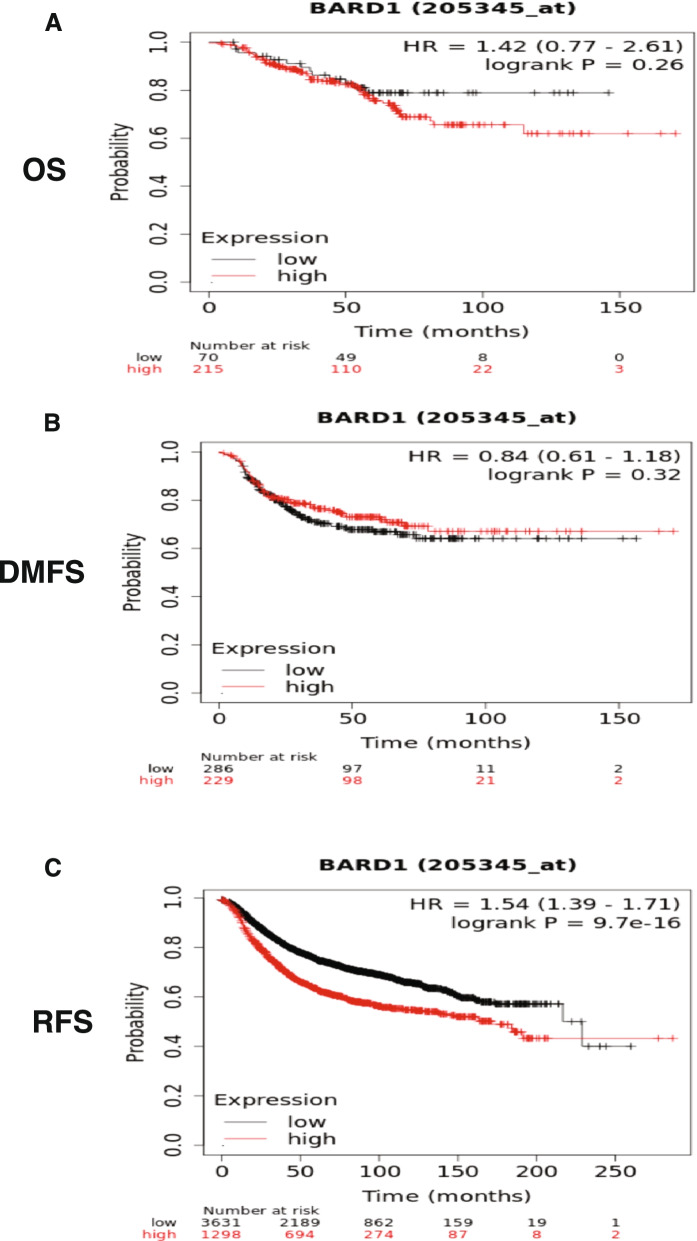
The Kaplan Meier plotter shows the survival curve in breast cancer patients: **A** & **B** No significant correlation between the high expression of BARD1 gene and the overall survival (OS), *P* = 0.26 and (**B**) distant metastases free survival (DMFS), *P* = 0.32 in breast cancer patients who treated with chemotherapy. **C** Represent the high expression of BARD1 gene is significantly associated with shorter relapse free survival (RFS), *P* = 9.7 × 10^− 16^. Source: The Kaplan-Meier Plotter, (https://kmplot.com/analysis/) [71]

These findings highlight the potential involvement of BARD1 in modulating the response of the breast cancer cells to chemotherapeutic agents. To further validate this perception, we used Kaplan-Meier (KM) plotter (https://kmplot.com/analysis/) [99], with specific selection criteria where we assessed the effect of BARD1 expression in a group of breast cancer patients who received chemotherapy. As revealed in Fig. 9A-C**,** while we didn’t find a significant correlation between BARD1 expression and OS (***P*** = 0.26) or DMFS (***P*** = 0.32) in the selected patients interestingly high expression of BARD1 mRNA correlated with shorter RFS (***P*** = 9.7 × 10 ^− 16^**)** in the examined patients. This data suggests the negative / unfavourable impact of BARD1 expression on the response of breast cancer patients to cytotoxic agents. Indeed, these treated patients with high expression of BARD1 exhibit potential risk of disease relapse/ recurrence at one time in their life.

## Conclusions

**Fig. 10:**
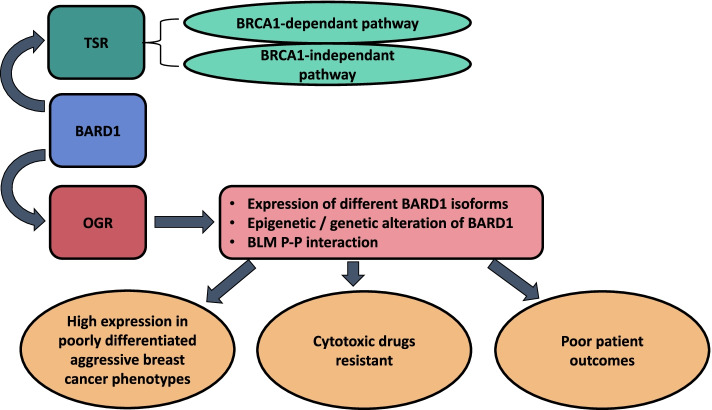
Schematic diagram of BARD1 dual roles in breast cancer: Diagram shows the dual roles of BARD1 gene in breast cancer. BARD1 (mainly the full length) displays a tumor suppressor role (TSR) by two mechanisms: BRCA1-dependant pathway and BRCA1-independent pathway. BARD1isoforms, epigenetic and or mutational events, or through protein-protein interaction with BLM demonstrated oncogenic role (OGR) during cancer progression associated with poor prognosis

The advancement of the oncological field is based on the comprehensive understanding of genetic background and unearthing of genetic aberrations that are involved in diseases' evolution and progression. In this study, we discussed plenteous reports that elucidated the dual roles of the BARD1 in cancer development (Fig. 10). BRCA1 and BARD1 proteins display many functional and structural criteria and thus BRCA1-BARD1 complex plays an essential role in maintaining genetic stability. Based on Structural and functional analysis of BARD1 gene as well as evidence from literature and bioinformatics database we concluded the BARD1 gene might potentially harbor a substantial engagement in breast cancer development and progression based on the presenting varient/s. Additionally, the BARD1 gene showed a contribution to the modulation of therapeutic effects of various treatment modalities. Further, we assessed different aspects of the BARD1 gene including its expression profile in cancers, mainly breast cancer, prognostic survival value, and patients’ outcome, and critical interaction with other proteins. Nevertheless, additional studies and practical investigations are required to elaborate on the role of BARD1 in cancer and assist in generating strategies to guide the tract of the oncology field.

We also aimed to attract attention to the importance of genetic screening for BARD1 and its isoforms. The introduction of a clinical test to detect the oncogenic isoforms of BARD1 would facilitate the early diagnosis of high-risk patients. This pointed to a new avenue in the treatment of breast cancer by using the BARD1 gene as a potential therapeutic and diagnostic target. Moreover, targeting blockers against these oncogenic isoforms might eventually be offered a positive impact on the efficiency of breast cancer therapy. A promising area of science that connect molecular biology to imaging medicine identified as radio genomics holds hope in cancer field management [39]. Finally, the BARD1 gene could offer a new avenue for advancing the field of breast cancer therapy.

## Data Availability

All data generated or analysed during this study are included in this published article.
